# The complete mitochondrial genome of *Halichoeres margaritaceus* (Actinopterygii, Labridae)

**DOI:** 10.1080/23802359.2025.2485160

**Published:** 2025-03-31

**Authors:** Xin Huang, Qi Zhou, Yiheng Xu, Qing Dong, Shitao Fang, Xiao Li, Yan Wu, Xunchao Cai

**Affiliations:** ^a^ Key Lab. of Biodiversity Conservation and Characteristic Resource Utilization in Southwest Anhui, Anqing Forestry Technology Innovation Research Institute, School of Life Sciences, Anqing Normal University, Anqing, PR China; ^b^ Department of Gastroenterology and Hepatology, Shenzhen University General Hospital, Shenzhen University, Shenzhen, China

**Keywords:** Mitochondrial genome, *Halichoeres margaritaceus*, phylogenetic position, gene map

## Abstract

*Halichoeres margaritaceus* Valenciennes 1839 is a polygynous species of coral fish. We sequenced the complete mitochondrial genome of *H. margaritaceus*, and conducted a comprehensive analysis to reveal its characteristics. Results showed that the mitochondrial genome is a closed circular molecule comprising 16,710 bp. Phylogenetic analysis based on mitochondrial whole genome sequences revealed *H. margaritaceus* as a sister group of *H. ornatissimus, H. tenuispinis*, etc., with maximal support. This study presents, for the first time, the complete mitochondrial genome of *H. margaritaceus*, offering valuable insights that can aid in future research on the biodiversity and conservation management of this species.

## Introduction

*Halichoeres margaritaceus* Valenciennes 1839, a prevalent fish species within coral reef ecosystems, is recognized for its significant role as an ornamental fish (Kuwamura 2022). Taxonomically, it is classified under the order Perciformes, family Labridae, and subfamily Cheilininae (Mikami 2013). This species exhibits the typical body shape of a perciform fish and is widely distributed in tropical and subtropical marine regions. It commonly resides in coral reef habitats, typically in shallow marine areas (LaPlante 2017). The body coloration of this species is highly variable, undergoing distinct changes during its growth (Laplante 2015). As a carnivorous fish, *H. margaritaceus* primarily preys on invertebrates and often engages in cooperative foraging, thereby playing a crucial role in the trophic structure of coral reefs (Campbell et al. 2011). To date, no complete mitochondrial genome of *H. margaritaceus* had been documented. This study provides the first report of its complete mitochondrial genome, offering valuable insights for further research on the biodiversity and management of *Halichoeres* species. The management plan may encompass a series of measures, such as the establishment of no-fishing zones, imposition of fishing quotas, enhancement of fish population dynamics monitoring, and intensification of enforcement efforts, among others. However, specific management measures may need to be further refined and adjusted based on actual conditions and scientific research.

## Materials and methods

**Figure 1.**
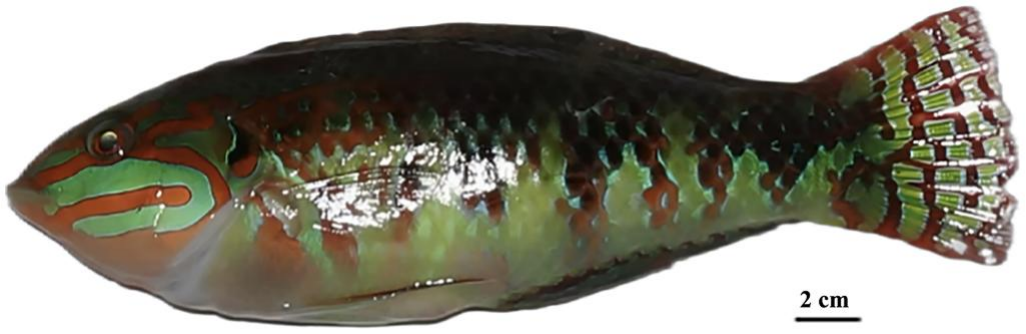
Photograph of *Halichoeres margaritaceus* (NS-YS-2022-8132). This photograph was taken by the first author (Xin Huang).

The freshly dead specimen of *H. margaritaceus* (Figure 1) was obtained from a local fisherman in Huangsha Aquatic Product Trading Market (23°7′50.34″N, 113°11′22.99″E), in December 2022. This study was approved by the Institutional Animal Care and Use Committee of the Anqing Normal University (Ref. AQNU-DWLL-20231102). Species identification was primarily based on the morphological characteristics, including features of the dorsal fin and the patterns of the pectoral and caudal fins. Then the muscle, fin, skin, venom gland and other visceral tissues were dissected and rinsed by autoclaved artificial seawater, followed by flash frozen in liquid nitrogen. The samples were preserved at −80 °C in the School of Life Sciences (https://smkx.aqnu.edu.cn/), Anqing Normal University (30°30′52.83″N, 117°2′56.33″E) under the voucher number NS-YS-2022-8132 (contact person: Xin Huang, email: 122307@aqnu.edu.cn) for future research applications.

Total DNA from pectoral fins was extracted using a column-based animal DNA extraction kit (Sun et al. 2022). Sequencing was performed using the Illumina NovaSeq 6000 platform (San Diego, CA, paired-end sequencing format, 33,812,497 raw reads with 150 bp length of each read). SPAdes (v3.11.1) (Bankevich et al. 2012) and NOVOPlasty (Dierckxsens et al. 2017) were used to assemble the mitochondrial genome, with the *Halichoeres tenuispinis* mitochondrial genome sequence (EU082205) serving as the reference, and the assembly yielded a circular contig. The complete mitogenome sequence was annotated using MitoAnnotator of MitoFish online tool (Iwasaki et al. 2013), with gene boundaries being manually verified for accuracy. The read depth (with approximately over 10× coverage) of the *H. margaritaceus* mitochondrial genome obtained through next-generation sequencing technology is shown in the supplementary materials (Figure S1).

Mitochondrial whole genome sequences were retrieved from GenBank, including all species of the genus Halichoeres (12 species) and some species of other related genera, by following two criteria: (1) ensured that all selected sequences were derived from fully sequenced and annotated mitochondrial genomes; (2) prioritized all species within the genus *Halichoeres* for which mitochondrial whole genome sequences were available and additionally included some closely related genera (Cowman et al. 2009). Furthermore, according to the literature (Matthew et al. 2018), *Epinephelus lanceolatus* (FJ472837.1) was selected as the outgroup to enhance the resolution and reliability of the phylogenetic tree. As such, a total of 20 species within the family Labridae, along with the outgroup sequence of *Epinephelus lanceolatus* (FJ472837.1), were selected to reconstruct their phylogenetic tree. The genome sequences from each species were aligned by ClustalW (Tamura et al. 2021). ModelFinder v2.2.0 (Kalyaanamoorthy et al. 2017) was employed to identify the most suitable evolutionary model (GTR+I + G). Subsequently, to construct a maximum likelihood (ML) phylogenetic tree using MEGA v11 with 1000 bootstrap replicates (Tamura et al. 2021). The MitoFish online tool (Zhu et al. 2023) was employed to generate the circular mitogenome maps. DNASTAR (Madison, WI) was used to determine the base composition of the complete mitochondrial genome (Burland 2000).

## Results

**Figure 2.**
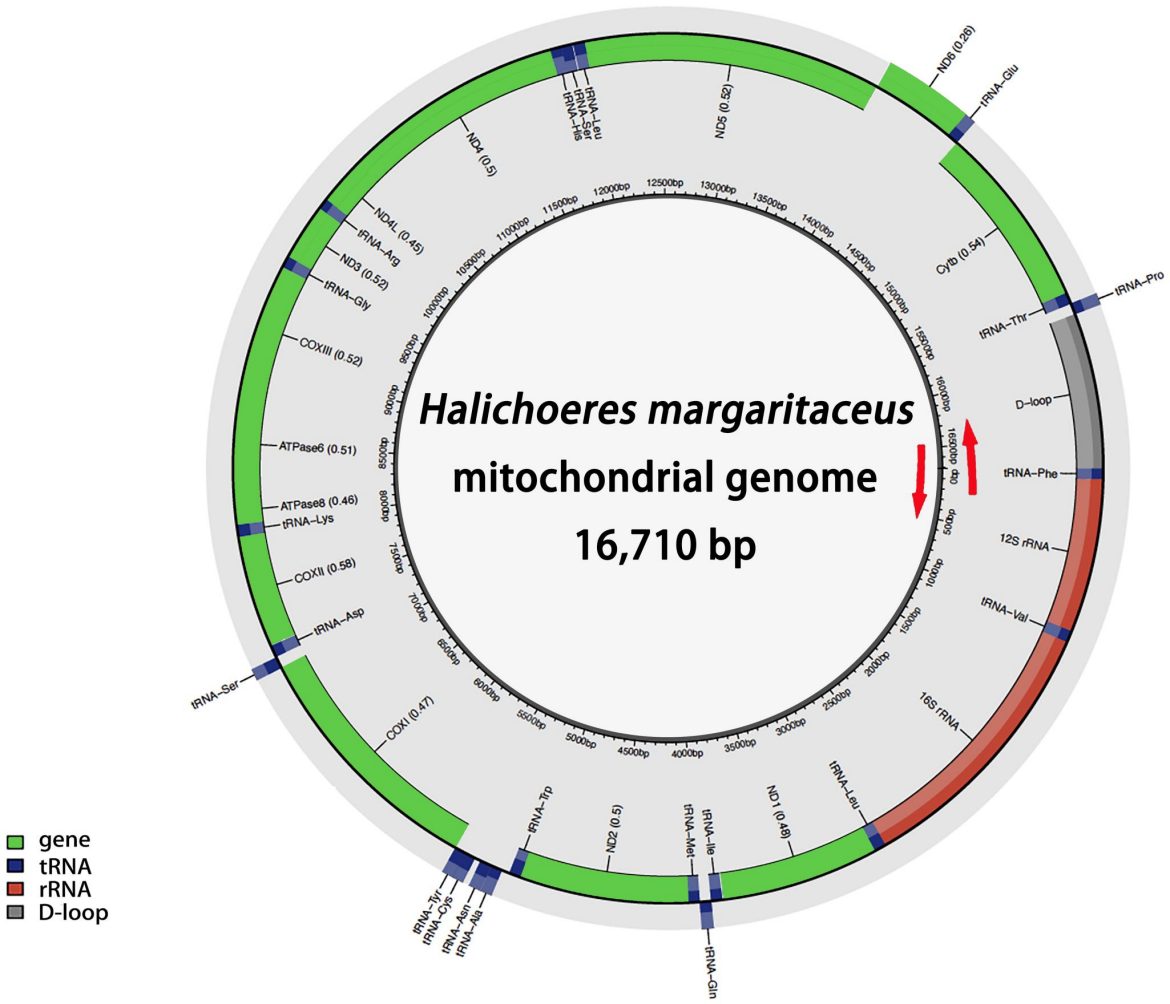
Gene map of the mitochondrial genome for *Halichoeres margaritaceus*. Red arrows indicate the orientation of gene transcription. Gene, rRNA, tRNA an D-loop are marked with different colors. The tRNAs are labeled according to the three-letter amino acid codes.

The sequencing and assembly analyses showed that the complete mitochondrial genome of *H. margaritaceus* is a closed circular molecule with a total length of 16,710 bp, carrying 13 protein-coding genes, 22 tRNA genes, two rRNA genes, and a major non-coding region (Figure 2, accession number PQ738625.1). The gene structure and sequence were in concordance with those observed in the mitochondrial genomes of Labridae fish species. The tRNA genes, including *trnG*, *trnA*, *trnN*, *trnC*, *trnY*, *trnS*, *trnE*, *trnP*, and *ND6* gene are encoded on the light chain, while other genes are encoded on the heavy chain. The A＋T content of the mitochondrial genome was determined to be 53.59%.

**Figure 3.**
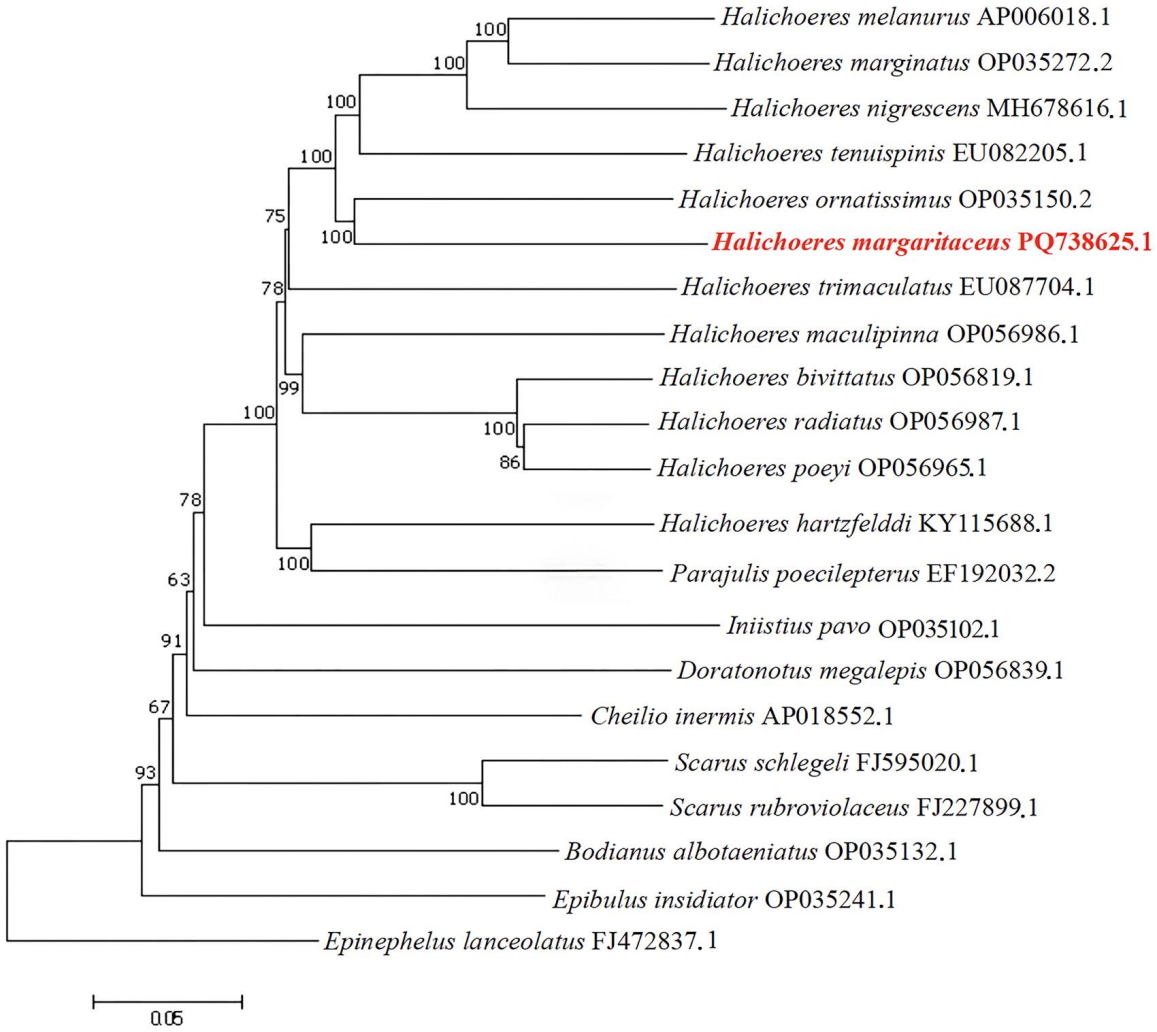
Maximum likelihood (ML) tree based on the mitochondrial whole genome sequences. The *Halichoeres margaritaceus* genome is marked in red bold font. GenBank accession numbers are provided after the scientific names, and follows: *Parajulis poecilepterus* EF192032.2 (Oh et al. 2007), *Halichoeres melanurus* AP006018.1 (Devadhasan et al. 2016), *Halichoeres ornatissimus* OP035150.2 (unpublished), *Halichoeres radiatus* OP056987.1 (unpublished), *Halichoeres poeyi* OP056965.1 (unpublished), *Halichoeres trimaculatus* EU087704.1 (Oh and Jung 2008), *Halichoeres hartzfeldii* KY115688.1 (Guo et al. 2019), *Halichoeres maculipinna* OP056986.1 (unpublished), *Halichoeres bivittatus* OP056819.1 (unpublished), *Halichoeres margaritaceus* PQ738625.1 (this study), *Halichoeres tenuispinis* EU082205.1 (Matthew et al. 2018), *halichoeres marginatus* OP035272.2 (unpublished), *Halichoeres nigrescens* MH678616.1 (Shi et al. 2018), *Iniistius pavo* OP035102.1 (unpublished), *Scarus schlegeli* FJ595020.1 (Gao et al. 2023), *Scarus rubroviolaceus* FJ227899.1 (Choi et al. 2020), *Doratonotus megalepis* OP056839.1 (unpublished), *Cheilio inermis* AP018552.1 (Song et al. 2018), *Epibulus insidiator* OP035241.1 (unpublished), *Bodianus albotaeniatus* OP035132.1 (unpublished), *Epinephelus lanceolatus* FJ472837.1 (Matthew et al. 2018).

Phylogenetic tree of Labridae family was reconstructed based on mitochondrial whole genome sequences using Maximum Likelihood (ML) method. As shown in the phylogenetic tree (Figure 3), *H. margaritaceus* was robustly identified as the sister group of *Halichoeres nigrescens*, *Halichoeres marginatis* and *Halichoeres temnispinis* with maximal support. This finding underscores the close phylogenetic affinity among these species within the *Halichoeres* genus, thereby validating their taxonomic cohesion. For a comprehensive analysis of phylogenetic trees, future in-depth studies should integrate other genetic markers, such as nuclear genomes or morphological traits, for an integrated evaluation.

## Discussion and conclusions

This study provides the first comprehensive analysis of the complete mitochondrial genome of *H. margaritaceus*, filling a critical gap in mitochondrial genomic data for this species. Through phylogenetic analysis, we found a strong sister relationship between *H. margaritaceus* and *H. ornatissimus* within the scope of our sampling, with a support value of 100% (Figure 3). Although using mitochondrial genomes to reflect species relationships has limitations, the phylogenetic results gleaned from this study are still resemble the traditional morphological classifications (Victor et al. 2024), further validating the value of mitochondrial genomes in species evolutionary research.

The A＋T content (53.59%) of the mitochondrial genome is consistent with the results reported for other vertebrates (Sun et al. 2020; Molina-Quirós et al. 2022), revealing the compositional conservation of vertebrate mitochondrial genomes. The coverage depth (Figure S1) is relatively uniform across the entire sequence, with no significant under-coverage or over-coverage regions observed, validating the reliability of the sequencing data. Moreover, the results of this study substantiated the monophyletic status of the genus *Halichoeres*, corroborating previous studies (Wainwright et al. 2018; Barber and Bellwood 2005). We plan to incorporate mitochondrial genome data from additional related species, such as *Thalassoma* and *Coris*, in future studies to expand the dataset and more comprehensively evaluate the diversity and evolutionary relationships of mitochondrial genomes.

In summary, this study not only provides valuable genetic resources for the genus *Halichoeres* but also lays a foundation for further research into the evolutionary history and phylogenetic status of coral reef fishes. Future studies can utilize these data in conjunction with additional genomic information to conduct comprehensive analyses of the evolutionary relationships among species within the family Labridae.

## Supplementary Material

HM_Supplemental material.docx

## Data Availability

The mitogenomic sequence data that support the findings of this study are openly available in the GenBank of NCBI at https://www.ncbi.nlm.nih.gov/ under accession no. PQ738625.1. The associated BioProject, SRA, and Bio-Sample numbers are PRJNA1197738, SRR31756740, and SAMN45814605, respectively.
