# Supplementary material for: The complete mitochondrial genome of Halichoeres margaritaceus (Actinopterygii, Labridae)
Source: Mitochondrial DNA B Resour. 2025 Mar 31;10(5):347–51. doi: 10.1080/23802359.2025.2485160 (PMC11960308; doi:10.1080/23802359.2025.2485160)
Supplement: HM_Supplemental material.docx [file TMDN_A_2485160_SM6307.docx]

**The complete mitochondrial genome of** ***Halichoeres margaritaceus* (Actinopterygii, Labridae)**

Xin Huang^1#^, Qi Zhou^1#^, Yiheng Xu^1^, Qin Dong^1^, Shitao Fang^1^, Xiao Li^1^, Yan Wu^1*^, Xunchao Cai^2*^

^1^Key Lab. of Biodiversity Conservation and Characteristic Resource Utilization in Southwest Anhui, Anqing Forestry Technology Innovation Research Institute, School of Life Sciences, Anqing Normal University, Anqing, 246133, Anhui Province, PR China.

^2^Department of Gastroenterology and Hepatology, Shenzhen University General Hospital, Shenzhen University, Shenzhen, China.

^#^Co-first authors: Xin Huang; Qi Zhou.

^*^Co-correspondence: Yan Wu, 2771264617@qq.com; Xunchao Cai, cxch1224@126.com.

**Abstract**

*Halichoeres margaritaceus* Valenciennes 1839, a polygynous coral fish, is primarily distributed in the Eastern Pacific and Western Indian Ocean. This species is renowned for its vibrant coloration, making it a popular choice for ornamental aquariums. Here, we sequenced the complete mitochondrial genome of *H. margaritaceus* utilizing next-generation sequencing technology, and conducted a comprehensive analysis to reveal its genomic characteristics. Results showed that the mitochondrial genome is a closed circular molecule comprising 16,710 bp, carrying 13 protein-coding genes, 22 tRNA genes, two rRNA genes, and a major non-coding region. The overall base composition of the mitochondrial genome is 27.52% A, 28.28% C, 26.07% T, and 18.13% G, with a high overall AT content (53.59%). Phylogenetic analysis based on concatenated amino acid sequences revealed *H. margaritaceus* as a sister group of *Halichoeres tenuispinis*, *Halichoeres* *marginatus* and *Halichoeres nigrescens* with maximal support, thereby reinforcing the monophyletic status of the genus *Halichoeres*. The results of this study proved the monophyly of the genus *Halichoeres*. Moreover, this study presents, for the first time, the complete mitochondrial genome of *H. margaritaceus*, offering valuable insights that can aid in future research on the biodiversity and conservation management of this species.

**Supplemental material**


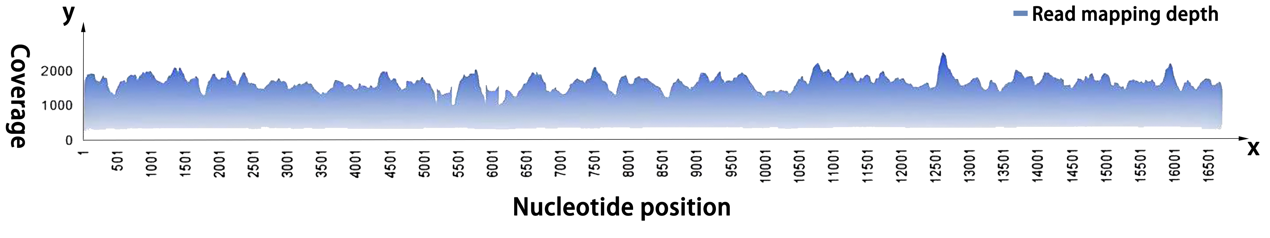


**Figure S1.** Read mapping depth of the complete mitochondrial genome sequence of *Halichoeres margaritaceus*. The x- and y-axes, nucleotide position and coverage, respectively. The blue section indicates the actual reading depth at each nucleotide position via the next-generation sequencing technology.
